# Neighborhood collective efficacy and environmental exposure to firearm homicide among a national sample of adolescents

**DOI:** 10.1186/s40621-023-00435-8

**Published:** 2023-06-09

**Authors:** Amanda J. Aubel, Angela Bruns, Xiaoya Zhang, Shani Buggs, Nicole Kravitz-Wirtz

**Affiliations:** 1 Violence Prevention Research Program, Department of Emergency Medicine, University of California Davis School of Medicine, 2315 Stockton Blvd, Sacramento, CA 95817 USA; 2 Department of Sociology and Criminology, Gonzaga University, 502 E Boone Ave, Spokane, WA 99258 USA; 3 Department of Family, Youth and Community Sciences, Institute of Food and Agricultural Sciences, University of Florida, 1604 McCarty Drive, PO Box 110310, Gainesville, FL 32611 USA

**Keywords:** Neighborhood collective efficacy, Firearm violence, Adolescents, Income, Race/ethnicity, Disparities

## Abstract

**Background:**

Living near an incident of firearm violence can negatively impact youth, regardless of whether the violence is experienced firsthand. Inequities in household and neighborhood resources may affect the prevalence and consequences of exposure across racial/ethnic groups.

**Findings:**

Using data from the Future of Families and Child Wellbeing Study and the Gun Violence Archive, we estimate that approximately 1 in 4 adolescents in large US cities lived within 800 m (0.5 miles) of a past-year firearm homicide during 2014–17. Exposure risk decreased as household income and neighborhood collective efficacy increased, though stark racial/ethnic inequities remained. Across racial/ethnic groups, adolescents in poor households in moderate or high collective efficacy neighborhoods had a similar risk of past-year firearm homicide exposure as middle-to-high income adolescents in low collective efficacy neighborhoods.

**Conclusions:**

Empowering communities to build and leverage social ties may be as impactful for reducing firearm violence exposure as income supports. Comprehensive violence prevention efforts should include systems-level strategies that jointly strengthen family and community resources.

**Supplementary Information:**

The online version contains supplementary material available at 10.1186/s40621-023-00435-8.

## Introduction

In recent years, nearly 20,000 people have died by firearm homicide annually in the USA, the highest death tolls ever recorded, and it is estimated that nearly three times as many people are treated in emergency departments for nonfatal assaultive firearm injuries (Centers for Disease Control and Prevention, National Center for Health Statistics 2023; Kaufman et al. 2021). Beyond fatal and nonfatal injuries, research estimates that 13% of youth aged 14–17 have heard gunshots or seen someone shot in their lifetime (Finkelhor et al. 2015). Both personal victimization and indirect exposure to violence have well-documented detrimental impacts on adolescents’ health and well-being and disproportionately affect low-income, urban-dwelling youth of color (Bancalari et al. 2022).

Theory and growing empirical evidence suggest that young people can also be affected by gun violence in their environment, regardless of whether they experience or witness it firsthand (Buggs et al. 2021; Gard et al. 2021; Heissel et al. 2018; Leibbrand et al. 2020; Sharkey 2010; Sharkey et al. 2012). This environmental firearm violence exposure, by which we mean living or spending time in places in which firearm violence has occurred, is even more widespread than direct or witnessed victimization. An estimated 1 in 5 adolescents in large US cities live or attend school within 500 m (0.3 miles) of a past-year firearm homicide, with stark inequities patterned by race/ethnicity, household poverty, and particularly concentrated neighborhood disadvantage (James et al. 2021; Kravitz-Wirtz et al. 2022). While it is critical to document and problematize these compounding adversities and their racialized consequences, a concern with so-called 'damage-centered research is that it is a pathologizing approach in which oppression singularly defines a community' (Tuck 2009: p.413). To effect change, research must also capture the deep knowledge, complexity, and agency that exists within marginalized communities and the ways in which community members resist, challenge, and disrupt the structural constraints that contribute to firearm violence.

To this end, previous research has found that strong social ties among neighbors and the organizational power of communities are associated with decreased rates of violence and may mitigate the negative impacts of violence exposure on youth (Fagan et al. 2014; Browning et al. 2014). In their seminal study of Chicago neighborhoods, Sampson and colleagues (1997) found that neighborhood collective efficacy—defined as social cohesion and mutual trust among neighbors (‘social cohesion’) combined with their willingness to intervene for the common good (‘informal social control’)—was associated with decreases in multiple measures of violence and largely mediated the relationship between neighborhood structural conditions and violence. These results have since been replicated in additional cities and countries (Burchfield and Silver 2013; Armstrong et al. 2015; Zhang et al. 2007; Mazerolle et al. 2010), lending support to notions that building collective efficacy may be an effective mechanism for preventing community violence and responding to other structurally rooted health problems that disproportionately burden low-income and minoritized communities.

However, few studies of neighborhood collective efficacy have examined firearm violence specifically, let alone broader environmental exposure to fatal firearm violence. As an initial step toward addressing this gap, the current study uses a general population sample of adolescents in large US cities merged with uniquely fine-grained, incident-level firearm violence data to estimate the prevalence and predicted probability of environmental exposure to deadly firearm violence at the nexus of race/ethnicity, household income, and neighborhood collective efficacy. Findings can help leverage strategies rooted in community strengths that may protect youth, and particularly low-income and minoritized youth of color, from firearm violence exposure and its harmful effects.

## Methods

### Data

Data come from the Future of Families and Child Wellbeing Study (FFCWS) and the Gun Violence Archive (GVA). The FFCWS is a probability-based birth cohort study following a nationally representative sample of 3442 families with children born in 1998–2000 in 16 randomly selected US cities with populations of 200,000 or more. Data are currently available across 6 waves of follow-up. Wave 6 was conducted between 2014 and 2017 when focal children were approximately 15 years of age. The GVA is a national, open-source database that has provided near real-time data on gun violence incidents, including their timing and location, since 2014; such fine-grained data on violence and crime are not otherwise reported on a national level. Data from the GVA and FFCWS-Wave 6 have been spatiotemporally linked by FFCWS staff, generating cross-classified measures of fatal firearm violence within various distances from adolescents’ homes (ranging from 100 to 1 mile) and various time periods before their Wave 6 interview date (ranging from 7 days to 1 year).

### Measures

Adolescents’ *environmental exposure to firearm homicide* was measured as a dichotomous variable indicating whether at least 1 firearm homicide had occurred within 800 m of the adolescent’s home in the past 365 days.^1^ We chose 800 m (0.5 miles) to align with the estimated radius of a median-sized US neighborhood (Donaldson 2013).

Adolescents’ *race/ethnicity* was self-reported and classified into four categories: non-Hispanic Black, Hispanic/Latinx, non-Hispanic white, and non-Hispanic other or multiracial. Consistent with prior research using this sample, mothers’ self-reported race/ethnicity was substituted when youths’ own reports were unknown or missing (James et al. 2021; Kravitz-Wirtz et al. 2022).

*Household income* was measured as the ratio of total household income to the prior year federal poverty level (FPL) established by the US Census Bureau and grouped into 3 categories: poor (< 100% FPL), near poor (100–199% FPL), and middle-to-high income (200% + FPL).^2^

*Neighborhood collective efficacy* was based on 9 items answered by adolescents’ primary caregivers (PCGs), adapted from Sampson and colleagues’ (1997) ‘social cohesion’ and ‘informal social control’ subscales; each item was rated on a 4-point Likert-type scale (see Methods in Additional file 1). Consistent with previous research using the FFCWS, we computed collective efficacy scores by averaging individual responses across items and then divided these scores into tertiles representing low, moderate, and high collective efficacy (Burdette et al. 2006; Kimbro and Schachter 2011). Individuals missing 1 or more item(s) were excluded from main analyses.

### Analysis

The analytic sample for this study included 1736 of 2494 (70%) adolescents who completed a FFCWS-Wave 6 interview; their sociodemographic characteristics are described in Table 1. We excluded adolescents who were interviewed in 2014 and, thus, had less than 1 year of GVA data (*n* = 431) or those who were missing data on their home address (*n* = 23), household income (*n* = 17), or neighborhood collective efficacy (*n* = 287). We calculated descriptive statistics (weighted percentages and their 95% confidence intervals [CI]) for adolescents’ environmental exposure to firearm homicide, overall and by race/ethnicity, household income, and neighborhood collective efficacy. We then used weighted logistic regression to calculate the predicted probability (or “risk”) of past-year exposure for adolescents in each cross-classification of race/ethnicity, household income, and neighborhood collective efficacy. When weighted, estimates are designed to be statistically representative of youth born in large US cities between 1998 and 2000.

**Table 1:** Sociodemographic characteristics of analytic sample of adolescents (*n* = 1736)

Characteristic	Unweighted *N*	Weighted % (95% CI)
*Age*		
14	8	0.08 (0.00–82.79)
15	1021	79.40 (67.85–87.56)
16	550	18.75 (11.61–28.84)
17	125	1.66 (0.94–2.93)
18	30	0.11 (0.04–0.30)
*Sex*		
Male	916	56.33 (49.37–63.04)
Female	820	43.67 (36.96–50.63)
*Primary caregiver*		
Biological mother	1497	90.17 (86.69–92.81)
Biological father	148	7.50 (4.91–11.28)
Non-parental caregiver	91	2.34 (1.74–3.13)
*Race/ethnicity*		
Black, non-Hispanic	705	23.32 (16.48–31.92)
Latinx/Hispanic	514	30.92 (22.43–40.92)
Other/multiracial, non-Hispanic	138	8.82 (5.59–13.64)
White, non-Hispanic	379	36.94 (31.62–42.60)
*Household income*		
Poor (< 100% FPL)	477	22.13 (17.10–28.14)
Near poor (100–199% FPL)	478	21.73 (16.21–28.50)
Middle-to-high income (200% + FPL)	781	56.14 (48.48–63.51)
*Neighborhood collective efficacy*		
Low	536	30.83 (24.56–37.90)
Moderate	583	34.10 (28.26–40.46)
High	617	35.07 (28.77–41.94)

*FPL* federal poverty level

We conducted two supplementary analyses to reduce missingness and test the robustness of our predicted probabilities. First, instead of excluding individuals missing any collective efficacy items, we included adolescents whose PCG answered more than 50% of items in each of the two subscales, averaging their responses on the completed items only (*n* = 1888). Second, we included adolescents who were interviewed in 2014 and, thus, had incomplete firearm homicide exposure data (*n* = 2161). Findings from these analyses were substantively similar to our main findings and can be found in Additional file 1: Tables S1 and S2.

Given prior research documenting differences in parents’ and their adolescent children’s perceptions of neighborhood violence and collective efficacy (Johnson et al. 2011), we also computed collective efficacy scores using adolescent self-reports instead of their PCG (*n* = 1829); adolescents were asked 8 of the 9 collective efficacy items (see Methods in Additional file 1). Collective efficacy scores for adolescents and their PCGs were only weakly correlated (*r* = 0.26), and less than half (42.70%) of dyads were classified in the same tertile (Additional file 1: Table S3). The median score was lower for PCGs than adolescents, indicating higher perceived collective efficacy among PCGs (Additional file 1: Fig. S1). Findings from this analysis are in Additional file 1: Table S4 and Fig. S2 and discussed below.

All analyses were conducted in Stata, version 16.1 (StataCorp LP, College Station, TX, USA). This study of secondary data was deemed exempt from human subjects review by the Institutional Review Boards at the University of California, Davis, and Gonzaga University.

## Results

### Prevalence of firearm homicide exposure

Approximately one quarter (24.40%; 95% CI: 20.74–28.47) of adolescents experienced a past-year firearm homicide within 800 m of their home (Table 2). Prevalence was higher among Black (36.06%; 95% CI: 29.66–43.01), Latinx (33.50%; 95% CI: 23.42–45.35), and other/multiracial youth (29.77%; 95% CI: 13.36–53.80) compared with white peers (8.15%; 95% CI: 4.30–14.90), and decreased with increasing household income (from 41.99% [95% CI: 30.30–54.65] in poor households to 12.62% [95% CI: 8.17–18.99] in middle-to-high-income households) and neighborhood collective efficacy (from 42.14% [95% CI: 32.07–52.90] in low to 13.68% [95% CI: 9.91–18.59] in high collective efficacy neighborhoods).

**Table 2:** Percentage of adolescents exposed to firearm homicide within 800 m of their home in the past year, total and by race/ethnicity, household income and neighborhood collective efficacy (*n* = 1736)

Characteristic	Unweighted *N*	Weighted % (95% CI)
Total, exposed to firearm homicide	512	24.40 (20.74–28.47)
*Race/ethnicity*		
Black, non-Hispanic	316	36.06 (29.66–43.01)
Latinx/Hispanic	131	33.50 (23.42–45.35)
Other/multiracial, non-Hispanic	36	29.77 (13.36–53.80)
White, non-Hispanic	29	8.15 (4.30–14.90)
*Household income*		
Poor (< 100% FPL)	206	41.99 (30.30–54.65)
Near poor (100–199% FPL)	157	36.93 (21.64–55.39)
Middle-to-high income (200% + FPL)	149	12.62 (8.17–18.99)
*Neighborhood collective efficacy*		
Low	218	42.14 (32.07–52.90)
Moderate	156	19.39 (12.59–28.66)
High	138	13.68 (9.91–18.59)

*FPL* federal poverty level

### Predicted probability of firearm homicide exposure at the intersection of neighborhood collective efficacy, household income, and race/ethnicity

**Fig. 1:**
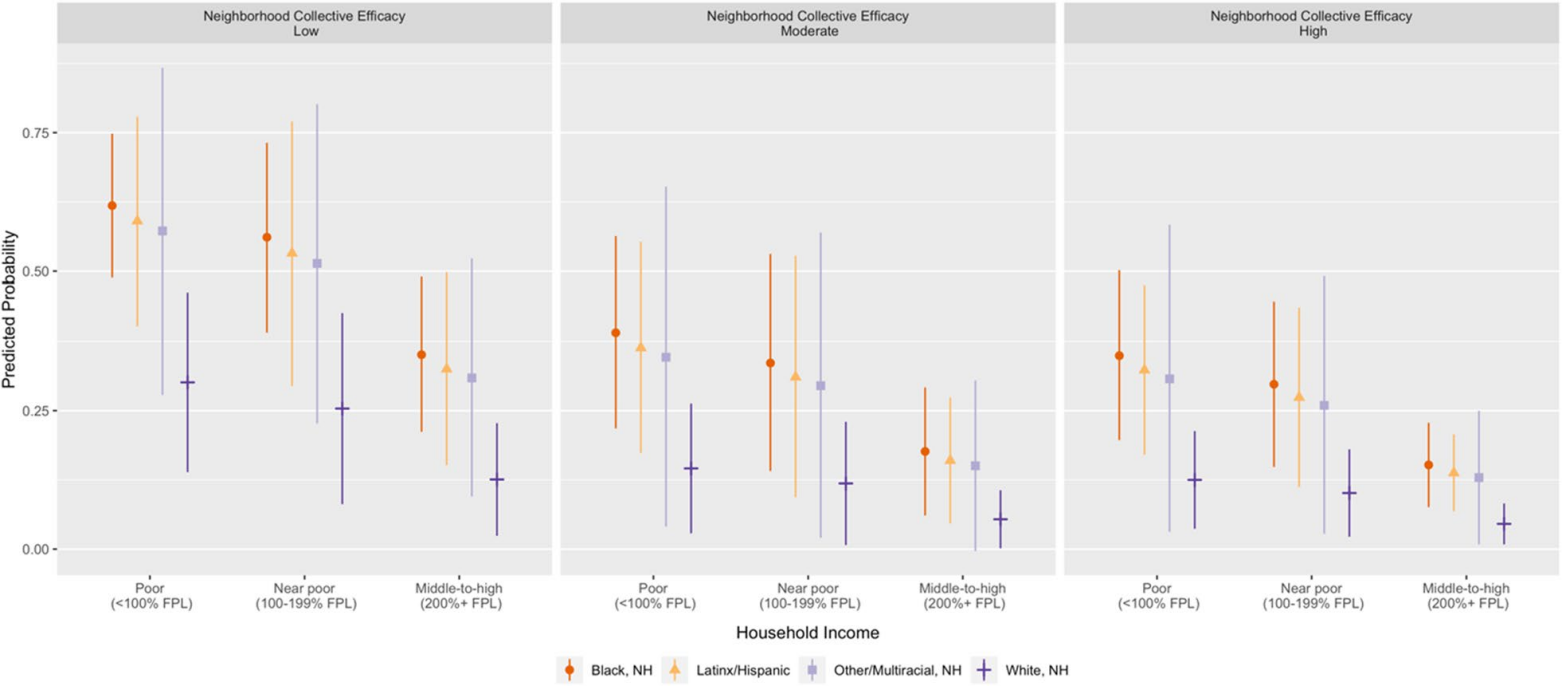
Predicted probability of adolescents’ past-year exposure to firearm homicide within 800 m of their home by race/ethnicity, household income & neighborhood collective efficacy (*n* = 1736)

Taken together, the predicted probability of firearm homicide exposure was highest among youth in poor households in low collective efficacy neighborhoods, ranging from 30.10% (95% CI: 13.90–46.30) for White youth to 61.83% (95% CI: 48.85–74.81) for Black youth, and lowest among those in middle-to-high-income households in high collective efficacy neighborhoods, ranging from 4.55% (95% CI: 0.85–8.24) for White youth to 15.19% (95% CI: 7.59–22.08) for Black youth (Fig. 1 and Additional file 1: Table S5).

Across racial/ethnic-income groups, exposure risk decreased as neighborhood collective efficacy increased, with relatively larger differences between low and moderate collective efficacy neighborhoods (7 to 23 percentage points) than between moderate and high (1 to 4 percentage points). By extension, adolescents in middle-to-high-income households in low collective efficacy neighborhoods had nearly equivalent risk of firearm homicide exposure as those in poor households in either moderate or high collective efficacy neighborhoods, though racial/ethnic inequities remained. In contrast, when using adolescents’ reports of neighborhood collective efficacy (vs. their PCGs’) in supplementary analyses, the probability of firearm homicide exposure within racial/ethnic-income groups remained relatively stable across levels of collective efficacy (Additional file 1: Fig. S2 and Table S4).

## Discussion

This study adds to the growing literature on adolescents’ environmental exposure to firearm violence and suggests that neighborhoods where residents share norms and values, trust one another, and are empowered to intervene to address problems (i.e., higher collective efficacy) may provide some level of protection for youth, even in the context of household socioeconomic disadvantage. National studies have previously estimated that 5% of youth aged 14–17 have witnessed a shooting in the past year (Finkelhor et al. 2015). We estimate that far more young people—nearly 1 in 4 in large US cities—had a past-year firearm homicide that occurred within 800 m, or just a 10-min walk, from their home. However, risk of environmental firearm homicide exposure decreased by approximately half for the lowest income youth living in high versus low collective efficacy neighborhoods, across all racial/ethnic groups. Notably, the greatest reductions in risk were observed between low versus moderate collective efficacy neighborhoods (rather than moderate vs. high), especially for low-income youth of color, suggesting that large gains in health and safety can be achieved through relatively modest efforts to leverage social ties in the most structurally disadvantaged neighborhoods.

Scholarship on community firearm violence often focuses on the role of historical and contemporary forms of oppression in perpetuating racial/ethnic inequities in firearm violence exposure and its associated harms (Kravitz-Wirtz et al. 2022; Poulson et al. 2021). While it is important to recognize and respond to the structural and racist underpinnings of, and interrelationships between, concentrated disadvantage and firearm violence in marginalized and minoritized communities, it is also important to acknowledge the power and strengths within communities to inform strategies for reducing harm. For example, we found that, across racial/ethnic groups, low-income youth in either moderate or high collective efficacy neighborhoods had approximately the same probability of firearm homicide exposure as middle-to-high income youth in low collective efficacy neighborhoods. Thus, reinforcing and expanding existing relationships between neighbors and incorporating meaningful opportunities to leverage those ties for community benefit may be as impactful for reducing firearm violence (exposure) as the well-known advantages associated with greater socioeconomic resources (Rowhani-Rahbar et al. 2022).

Interestingly, when using adolescents’ own reports of neighborhood collective efficacy, which were weakly correlated with their PCGs’, there was little to no variation in the probability of firearm homicide exposure between levels of collective efficacy. Previous research has likewise observed inconsistencies between adolescents’ and their parents’ perceptions of collective efficacy and in their associations with youth violence exposure and outcomes (Gard et al. 2021; Fagan et al. 2014; Johnson et al. 2011). Future research should further investigate these differences, as well as how perceived neighborhood collective efficacy relates to actual social ties, informal social control behavior, and community firearm violence (Magee 2020).

Additional research is also needed to better understand how neighborhood collective efficacy can be fostered, including, for example, through safe and inclusive community centers, improvements to the built environment (e.g., parks, outdoor spaces, sidewalks), organized community groups and activities (e.g., volunteering, cultural activities, arts projects, sports), or collective efficacy training programs (Breedvelt et al. 2022; Ohmer 2016). Community-based violence intervention programs, which utilize credible messengers who work to disrupt violence and are well-known to the communities they serve, may also facilitate collective efficacy by building relationships with community members, shifting community norms, and organizing community-based peacebuilding events (Corburn et al. 2021). However, programs and community members alike need access to institutional supports, including adequate funding for such supportive services, linkages with established neighborhood organizations and external resources from public sector organizations (e.g., public safety, government), to develop collective efficacy and sustain violence prevention efforts (Ohmer 2016).

### Limitations

This study has limitations. First, our results are primarily descriptive and are not intended to establish causality, directionality, or statistical significance. Second, we are likely underestimating the full scope of adolescents’ environmental exposure to firearm violence because data on nonfatal shootings were not available and we used a dichotomous measure of firearm homicide exposure. Questions remain about whether and how neighborhood collective efficacy affects the risk, or mitigates the effects, of repeated exposure to fatal and nonfatal firearm violence, in both unadjusted and more fully adjusted analyses. Third, while our estimates are weighted to statistically represent youth born in large US cities between 1998 and 2000, they may not generalize to youth in rural areas or smaller cities or to youth born in different time periods

## Conclusion

Environmental exposure to firearm violence is common and unequally distributed among adolescents in the USA, reflecting the concentration of structural disadvantage in minoritized communities. Although household socioeconomic status contributes to these inequities, cohesive and trusting community relationships and the capacity for collective action may additionally mitigate firearm violence risk. Given well-documented consequences of firearm violence exposure on the health and well-being of young people, comprehensive violence prevention strategies should not only increase the resilience of individuals and families through income support policies, but also build on neighborhood strengths by fostering collective efficacy.

## Supplementary Information

**Additional file 1**. Neighborhood collective efficacy measures and supplementary tables and figures.

## Data Availability

The data that support the findings of this study are available from https://ffcws.princeton.edu/, but restrictions apply to the availability of these data. The authors do not have permission to share data.

## Abbreviations

FFCWS: Future of Families and Child Wellbeing Study
FPL: Federal poverty level
GVA: Gun Violence Archive
PCG: Primary caregiver
US: United States

## Footnotes

1 We use the term firearm homicide to describe the fatal firearm violence incidents in the linked FFCWS-GVA dataset. While the dataset may also include unintentional and undetermined firearm deaths, these made up approximately 2.1% of annual firearm deaths in the USA, on average, during the data collection period (2014–2017).

2 We used the same categories and labels for household income as James et al. (2021) for consistency and for lack of better terms. The median household income was $15,000 (IQR: $8,040-$23,000) for poor households, $37,247 (IQR: $30,000-$50,000) for near poor households, and $91,245 (IQR: $68,000-$133,027) for middle-to-high-income households in our analytic sample. For comparison, the median household income for all US households according to the 2015 American Community Survey was $55,775.
