# Supplementary material for: Neighborhood collective efficacy and environmental exposure to firearm homicide among a national sample of adolescents
Source: Inj Epidemiol. 2023 Jun 9;10:24. doi: 10.1186/s40621-023-00435-8 (PMC10251689; doi:10.1186/s40621-023-00435-8)
Supplement: Supplementary file 1 — Additional file 1. Neighborhood collective efficacy measures and supplementary tables and figures. [file 40621_2023_435_MOESM1_ESM.docx]

**Supplementary material for "Neighborhood collective efficacy and environmental exposure to firearm homicide among a national sample of adolescents”**

**Amanda J. Aubel, Angela Bruns, Xiaoya Zhang, Shani Buggs, and Nicole Kravitz-Wirtz**

**Methods.** Neighborhood collective efficacy items from the Future of Families and Child Wellbeing Study (FFCWS), Wave 6 Teen and Primary Caregiver Interview………………………………………….Page 1

**Table S1.** Predicted probability of adolescents’ past-year exposure to firearm homicide within 800 meters of their home by race/ethnicity, household income & neighborhood collective efficacy (n=1,888)………

………………………………………………………………………………………………………..Page 2

**Table S2.** Predicted probability of adolescents’ past-year exposure to firearm homicide within 800 meters of their home by race/ethnicity, household income & neighborhood collective efficacy (n=2,161) ………

………………………………………………………………………………………………………..Page 3

**Table S3.** Crosstabulation of neighborhood collective efficacy tertiles based on primary caregivers’ reports and adolescents’ reports (n=1,626)………………………………………………………….Page 4

**Figure S1a.** Distribution of primary caregivers’ collective efficacy scores (n=1,736) ……………Page 5

**Figure S1b.** Distribution of adolescents’ collective efficacy scores (n=1,829) ….………………...Page 5

**Table S4.** Predicted probability of adolescents’ past-year exposure to firearm homicide within 800 meters of their home by race/ethnicity, household income & adolescents’ reports of neighborhood collective efficacy (n=1,829) …………………………………………………………………………………..Page 6

**Figure S2.** Predicted probability of adolescents’ past-year exposure to firearm homicide within 800 meters of their home by race/ethnicity, household income & adolescents’ reports of neighborhood collective efficacy (n=1,829)………………………………………………………………………...Page 7

**Table S5.** Predicted probability of adolescents’ past-year exposure to firearm homicide within 800 meters of their home by race/ethnicity, household income & neighborhood collective efficacy (n=1,736) ……………………………………………………………………………………………Page 8

Methods. Neighborhood collective efficacy items from the Future of Families and Child Wellbeing Study (FFCWS), Wave 6 Teen and Primary Caregiver Interview

*Social cohesion and trust*

Now I’m going to read some statements about your neighborhood and the people who live there. For each statement, please tell me if you strongly agree, somewhat agree, somewhat disagree, or strongly disagree.

|  | Strongly agree (1) | Somewhat agree  (2) | Somewhat disagree (3) | Strongly disagree (4) | Refused | Don’t know |
| --- | --- | --- | --- | --- | --- | --- |
| People around here are willing to help their neighbors |  |  |  |  |  |  |
| This is a close-knit neighborhood |  |  |  |  |  |  |
| People in this neighborhood generally don’t get along with each other* |  |  |  |  |  |  |
| People in this neighborhood do not share the same values* |  |  |  |  |  |  |

*Reverse-coded

*Informal social control*

For each item I read, please tell me whether your neighbors would be very likely, somewhat likely, not very likely, or very unlikely to do something or get involved.

|  | Very likely  (1) | Somewhat likely  (2) | Not very likely  (3) | Very unlikely (4) | Refused | Don’t know |
| --- | --- | --- | --- | --- | --- | --- |
| If children were skipping school and hanging out on the street |  |  |  |  |  |  |
| If children were spray painting buildings with graffiti |  |  |  |  |  |  |
| If children were showing disrespect to an adult |  |  |  |  |  |  |
| If a fight broke out in front of the house or building |  |  |  |  |  |  |
| If the fire station closest to the neighborhood was threatened and its budget was cut** |  |  |  |  |  |  |

**Only asked of primary caregivers

Table S1. Predicted probability of adolescents’ past-year exposure to firearm homicide within 800 meters of their home by race/ethnicity, household income & neighborhood collective efficacy (n=1,888)*

| Income x Collective efficacy | Race/ethnicity | Coefficient | 95% CI | |
| --- | --- | --- | --- | --- |
|  |  |  | Lower | Upper |
| Poor-  Low collective efficacy | Black, Non-Hispanic | 0.6163 | 0.4909 | 0.7418 |
|  | Latinx/Hispanic | 0.5765 | 0.4007 | 0.7523 |
|  | Other/Multiracial, Non-Hispanic | 0.5496 | 0.2694 | 0.8299 |
|  | White, Non-Hispanic | 0.2675 | 0.1217 | 0.4133 |
| Poor-  Moderate collective efficacy | Black, Non-Hispanic | 0.4028 | 0.2414 | 0.5643 |
|  | Latinx/Hispanic | 0.3638 | 0.1852 | 0.5423 |
|  | Other/Multiracial, Non-Hispanic | 0.3389 | 0.0537 | 0.6241 |
|  | White, Non-Hispanic | 0.1330 | 0.0274 | 0.2386 |
| Poor-  High collective efficacy | Black, Non-Hispanic | 0.3470 | 0.1981 | 0.4960 |
|  | Latinx/Hispanic | 0.3105 | 0.1734 | 0.4476 |
|  | Other/Multiracial, Non-Hispanic | 0.2876 | 0.0376 | 0.5377 |
|  | White, Non-Hispanic | 0.1078 | 0.0303 | 0.1853 |
| Near poor-  Low collective efficacy | Black, Non-Hispanic | 0.5570 | 0.3903 | 0.7237 |
|  | Latinx/Hispanic | 0.5159 | 0.2838 | 0.7480 |
|  | Other/Multiracial, Non-Hispanic | 0.4886 | 0.2162 | 0.7610 |
|  | White, Non-Hispanic | 0.2223 | 0.0640 | 0.3807 |
| Near poor-  Moderate collective efficacy | Black, Non-Hispanic | 0.3456 | 0.1530 | 0.5382 |
|  | Latinx/Hispanic | 0.3092 | 0.0928 | 0.5255 |
|  | Other/Multiracial, Non-Hispanic | 0.2863 | 0.0269 | 0.5458 |
|  | White, Non-Hispanic | 0.1072 | 0.0040 | 0.2104 |
| Near poor-  High collective efficacy | Black, Non-Hispanic | 0.2938 | 0.1511 | 0.4364 |
|  | Latinx/Hispanic | 0.2607 | 0.1102 | 0.4111 |
|  | Other/Multiracial, Non-Hispanic | 0.2402 | 0.0349 | 0.4454 |
|  | White, Non-Hispanic | 0.0864 | 0.0170 | 0.1558 |
| Middle-to-high income-  Low collective efficacy | Black, Non-Hispanic | 0.3646 | 0.2318 | 0.4975 |
|  | Latinx/Hispanic | 0.3272 | 0.1608 | 0.4936 |
|  | Other/Multiracial, Non-Hispanic | 0.3036 | 0.0914 | 0.5159 |
|  | White, Non-Hispanic | 0.1154 | 0.0275 | 0.2034 |
| Middle-to-high income-  Moderate collective efficacy | Black, Non-Hispanic | 0.1942 | 0.0827 | 0.3057 |
|  | Latinx/Hispanic | 0.1696 | 0.0568 | 0.2824 |
|  | Other/Multiracial, Non-Hispanic | 0.1548 | -0.0003 | 0.3098 |
|  | White, Non-Hispanic | 0.0519 | 0.0045 | 0.0994 |
| Middle-to-high income-  High collective efficacy | Black, Non-Hispanic | 0.1596 | 0.0766 | 0.2425 |
|  | Latinx/Hispanic | 0.1386 | 0.0654 | 0.2117 |
|  | Other/Multiracial, Non-Hispanic | 0.1261 | 0.0049 | 0.2472 |
|  | White, Non-Hispanic | 0.0414 | 0.0074 | 0.0754 |

*Sample includes adolescents whose primary caregivers answered more than 50% of collective efficacy items in each subscale (social cohesion and informal social control).

Table S2. Predicted probability of adolescents’ past-year exposure to firearm homicide within 800 meters of their home by race/ethnicity, household income & neighborhood collective efficacy (n=2,161)*

| Income x Collective efficacy | Race/ethnicity | Coefficient | 95% CI | |
| --- | --- | --- | --- | --- |
|  |  |  | Lower | Upper |
| Poor-  Low collective efficacy | Black, Non-Hispanic | 0.6247 | 0.5061 | 0.7434 |
|  | Latinx/Hispanic | 0.5882 | 0.4059 | 0.7705 |
|  | Other/Multiracial, Non-Hispanic | 0.5716 | 0.2837 | 0.8595 |
|  | White, Non-Hispanic | 0.3012 | 0.1458 | 0.4565 |
| Poor-  Moderate collective efficacy | Black, Non-Hispanic | 0.3993 | 0.2270 | 0.5716 |
|  | Latinx/Hispanic | 0.3632 | 0.1692 | 0.5572 |
|  | Other/Multiracial, Non-Hispanic | 0.3476 | 0.0468 | 0.6485 |
|  | White, Non-Hispanic | 0.1468 | 0.0337 | 0.2599 |
| Poor-  High collective efficacy | Black, Non-Hispanic | 0.3542 | 0.2061 | 0.5022 |
|  | Latinx/Hispanic | 0.3200 | 0.1689 | 0.4711 |
|  | Other/Multiracial, Non-Hispanic | 0.3054 | 0.0346 | 0.5762 |
|  | White, Non-Hispanic | 0.1243 | 0.0397 | 0.2089 |
| Near poor-  Low collective efficacy | Black, Non-Hispanic | 0.5666 | 0.4019 | 0.7313 |
|  | Latinx/Hispanic | 0.5287 | 0.2923 | 0.7651 |
|  | Other/Multiracial, Non-Hispanic | 0.5117 | 0.2347 | 0.7887 |
|  | White, Non-Hispanic | 0.2528 | 0.0858 | 0.4198 |
| Near poor-  Moderate collective efficacy | Black, Non-Hispanic | 0.3430 | 0.1437 | 0.5422 |
|  | Latinx/Hispanic | 0.3093 | 0.0859 | 0.5328 |
|  | Other/Multiracial, Non-Hispanic | 0.2950 | 0.0264 | 0.5636 |
|  | White, Non-Hispanic | 0.1190 | 0.0103 | 0.2278 |
| Near poor-  High collective efficacy | Black, Non-Hispanic | 0.3010 | 0.1525 | 0.4495 |
|  | Latinx/Hispanic | 0.2698 | 0.1065 | 0.4332 |
|  | Other/Multiracial, Non-Hispanic | 0.2566 | 0.0331 | 0.4802 |
|  | White, Non-Hispanic | 0.1003 | 0.0243 | 0.1763 |
| Middle-to-high income-  Low collective efficacy | Black, Non-Hispanic | 0.3553 | 0.2233 | 0.4873 |
|  | Latinx/Hispanic | 0.3211 | 0.1540 | 0.4881 |
|  | Other/Multiracial, Non-Hispanic | 0.3064 | 0.0946 | 0.5182 |
|  | White, Non-Hispanic | 0.1249 | 0.0245 | 0.2252 |
| Middle-to-high income-  Moderate collective efficacy | Black, Non-Hispanic | 0.1804 | 0.0669 | 0.2939 |
|  | Latinx/Hispanic | 0.1588 | 0.0463 | 0.2714 |
|  | Other/Multiracial, Non-Hispanic | 0.1500 | -0.0018 | 0.3018 |
|  | White, Non-Hispanic | 0.0539 | 0.0031 | 0.1047 |
| Middle-to-high income-  High collective efficacy | Black, Non-Hispanic | 0.1537 | 0.0811 | 0.2262 |
|  | Latinx/Hispanic | 0.1348 | 0.0680 | 0.2016 |
|  | Other/Multiracial, Non-Hispanic | 0.1271 | 0.0083 | 0.2458 |
|  | White, Non-Hispanic | 0.0449 | 0.0089 | 0.0809 |

*Sample includes adolescents who were interviewed in 2014 and had less than 1 year of data from the Gun Violence Archive.

Table S3. Crosstabulation of neighborhood collective efficacy tertiles based on primary caregivers’ reports and adolescents’ reports (n=1,626)

|  | Weighted % (Unweighted N) | | |
| --- | --- | --- | --- |
|  | Adolescents’ report | | |
| Primary caregivers’ report | High collective efficacy | Moderate collective efficacy | Low collective  efficacy |
| High collective efficacy | 19.19 (280) | 8.76 (167) | 7.30 (131) |
| Moderate collective efficacy | 11.38 (188) | 10.90 (194) | 10.95 (165) |
| Low collective efficacy | 10.17 (111) | 8.75 (186) | 12.61 (204) |

Figure S1a. Distribution of primary caregivers’ collective efficacy scores (n=1,736)

Note: Lower scores indicate higher collective efficacy.

Median = 1.67; Interquartile range = (1.22-2.11).

Figure S1b. Distribution of adolescents’ collective efficacy scores (n=1,829)

Note: Lower scores indicate higher collective efficacy.

Median = 2.13; Interquartile range = (1.63-2.50).

Table S4. Predicted probability of adolescents’ past-year exposure to firearm homicide within 800 meters of their home by race/ethnicity, household income & adolescents’ reports of neighborhood collective efficacy (n=1,829)

| Income x Collective efficacy | Race/ethnicity | Coefficient | 95% CI | |
| --- | --- | --- | --- | --- |
|  |  |  | Lower | Upper |
| Poor-  Low collective efficacy | Black, Non-Hispanic | 0.5176 | 0.3515 | 0.6836 |
|  | Latinx/Hispanic | 0.4924 | 0.2932 | 0.6917 |
|  | Other/Multiracial, Non-Hispanic | 0.4424 | 0.1738 | 0.7111 |
|  | White, Non-Hispanic | 0.1661 | 0.0491 | 0.2832 |
| Poor-  Moderate collective efficacy | Black, Non-Hispanic | 0.5026 | 0.3513 | 0.6539 |
|  | Latinx/Hispanic | 0.4775 | 0.3107 | 0.6443 |
|  | Other/Multiracial, Non-Hispanic | 0.4277 | 0.1258 | 0.7297 |
|  | White, Non-Hispanic | 0.1580 | 0.0316 | 0.2845 |
| Poor-  High collective efficacy | Black, Non-Hispanic | 0.4604 | 0.2898 | 0.6311 |
|  | Latinx/Hispanic | 0.4356 | 0.1737 | 0.6975 |
|  | Other/Multiracial, Non-Hispanic | 0.3869 | 0.0790 | 0.6949 |
|  | White, Non-Hispanic | 0.1368 | 0.0367 | 0.2369 |
| Near poor-  Low collective efficacy | Black, Non-Hispanic | 0.4699 | 0.3207 | 0.6190 |
|  | Latinx/Hispanic | 0.4450 | 0.2020 | 0.6879 |
|  | Other/Multiracial, Non-Hispanic | 0.3960 | 0.1808 | 0.6112 |
|  | White, Non-Hispanic | 0.1414 | 0.0319 | 0.2508 |
| Near poor-  Moderate collective efficacy | Black, Non-Hispanic | 0.4550 | 0.2722 | 0.6378 |
|  | Latinx/Hispanic | 0.4302 | 0.1807 | 0.6797 |
|  | Other/Multiracial, Non-Hispanic | 0.3818 | 0.1031 | 0.6605 |
|  | White, Non-Hispanic | 0.1342 | 0.0085 | 0.2600 |
| Near poor-  High collective efficacy | Black, Non-Hispanic | 0.4135 | 0.2052 | 0.6218 |
|  | Latinx/Hispanic | 0.3894 | 0.0716 | 0.7071 |
|  | Other/Multiracial, Non-Hispanic | 0.3428 | 0.0510 | 0.6345 |
|  | White, Non-Hispanic | 0.1158 | 0.0061 | 0.2254 |
| Middle-to-high income-  Low collective efficacy | Black, Non-Hispanic | 0.2751 | 0.1261 | 0.4241 |
|  | Latinx/Hispanic | 0.2555 | 0.1128 | 0.3982 |
|  | Other/Multiracial, Non-Hispanic | 0.2192 | 0.0537 | 0.3847 |
|  | White, Non-Hispanic | 0.0658 | 0.0111 | 0.1206 |
| Middle-to-high income-  Moderate collective efficacy | Black, Non-Hispanic | 0.2633 | 0.0920 | 0.4346 |
|  | Latinx/Hispanic | 0.2443 | 0.0956 | 0.3929 |
|  | Other/Multiracial, Non-Hispanic | 0.2091 | 0.0009 | 0.4173 |
|  | White, Non-Hispanic | 0.0623 | -0.0014 | 0.1260 |
| Middle-to-high income-  High collective efficacy | Black, Non-Hispanic | 0.2318 | 0.1352 | 0.3285 |
|  | Latinx/Hispanic | 0.2144 | 0.0698 | 0.3591 |
|  | Other/Multiracial, Non-Hispanic | 0.1825 | 0.0185 | 0.3465 |
|  | White, Non-Hispanic | 0.0531 | 0.0166 | 0.0896 |

Figure S2. Predicted probability of adolescents’ past-year exposure to firearm homicide within 800 meters of their home by race/ethnicity, household income & adolescents’ reports of neighborhood collective efficacy (n=1,829)

**
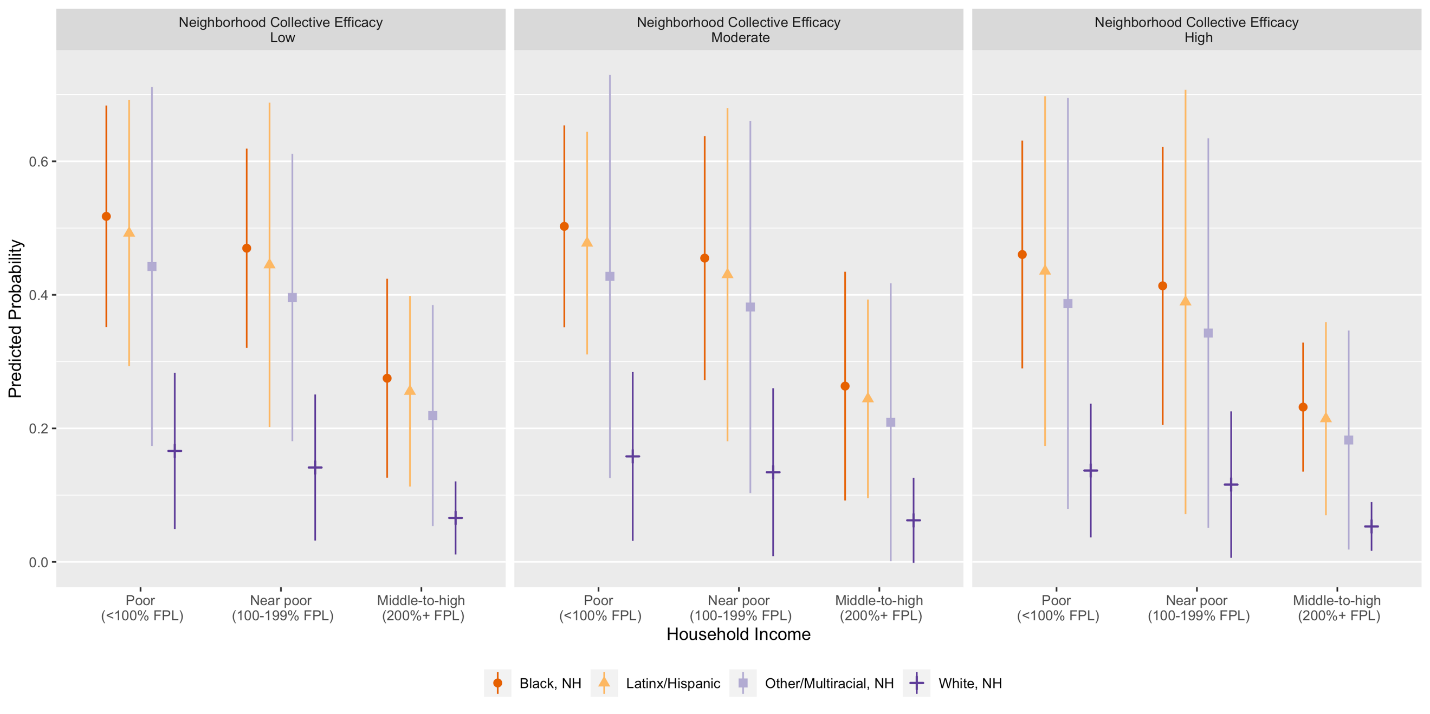
**

Table S5. Predicted probability of adolescents’ past-year exposure to firearm homicide within 800 meters of their home by race/ethnicity, household income & neighborhood collective efficacy (n=1,736)

| Income x Collective efficacy | Race/ethnicity | Coefficient | 95% CI | |
| --- | --- | --- | --- | --- |
|  |  |  | Lower | Upper |
| Poor-  Low collective efficacy | Black, Non-Hispanic | 0.6183 | 0.4885 | 0.7481 |
|  | Latinx/Hispanic | 0.5905 | 0.4022 | 0.7788 |
|  | Other/Multiracial, Non-Hispanic | 0.5726 | 0.2782 | 0.8671 |
|  | White, Non-Hispanic | 0.3010 | 0.1390 | 0.4630 |
| Poor-  Moderate collective efficacy | Black, Non-Hispanic | 0.3906 | 0.2179 | 0.5633 |
|  | Latinx/Hispanic | 0.3633 | 0.1736 | 0.5530 |
|  | Other/Multiracial, Non-Hispanic | 0.3465 | 0.0406 | 0.6523 |
|  | White, Non-Hispanic | 0.1456 | 0.0285 | 0.2627 |
| Poor-  High collective efficacy | Black, Non-Hispanic | 0.3492 | 0.1966 | 0.5018 |
|  | Latinx/Hispanic | 0.3233 | 0.1705 | 0.4760 |
|  | Other/Multiracial, Non-Hispanic | 0.3074 | 0.0310 | 0.5838 |
|  | White, Non-Hispanic | 0.1249 | 0.0367 | 0.2130 |
| Near poor-  Low collective efficacy | Black, Non-Hispanic | 0.5611 | 0.3907 | 0.7316 |
|  | Latinx/Hispanic | 0.5323 | 0.2945 | 0.7702 |
|  | Other/Multiracial, Non-Hispanic | 0.5140 | 0.2268 | 0.8012 |
|  | White, Non-Hispanic | 0.2537 | 0.0812 | 0.4262 |
| Near poor-  Moderate collective efficacy | Black, Non-Hispanic | 0.3360 | 0.1410 | 0.5310 |
|  | Latinx/Hispanic | 0.3105 | 0.0937 | 0.5274 |
|  | Other/Multiracial, Non-Hispanic | 0.2950 | 0.0204 | 0.5697 |
|  | White, Non-Hispanic | 0.1186 | 0.0074 | 0.2298 |
| Near poor-  High collective efficacy | Black, Non-Hispanic | 0.2975 | 0.1483 | 0.4468 |
|  | Latinx/Hispanic | 0.2738 | 0.1119 | 0.4357 |
|  | Other/Multiracial, Non-Hispanic | 0.2595 | 0.0278 | 0.4911 |
|  | White, Non-Hispanic | 0.1012 | 0.0223 | 0.1801 |
| Middle-to-high income-Low collective efficacy | Black, Non-Hispanic | 0.3510 | 0.2116 | 0.4903 |
|  | Latinx/Hispanic | 0.3250 | 0.1516 | 0.4983 |
|  | Other/Multiracial, Non-Hispanic | 0.3091 | 0.0951 | 0.5230 |
|  | White, Non-Hispanic | 0.1257 | 0.0239 | 0.2275 |
| Middle-to-high income-Moderate collective efficacy | Black, Non-Hispanic | 0.1763 | 0.0607 | 0.2918 |
|  | Latinx/Hispanic | 0.1600 | 0.0464 | 0.2736 |
|  | Other/Multiracial, Non-Hispanic | 0.1504 | -0.0038 | 0.3046 |
|  | White, Non-Hispanic | 0.0538 | 0.0014 | 0.1063 |
| Middle-to-high income-High collective efficacy | Black, Non-Hispanic | 0.1519 | 0.0759 | 0.2280 |
|  | Latinx/Hispanic | 0.1375 | 0.0683 | 0.2068 |
|  | Other/Multiracial, Non-Hispanic | 0.1291 | 0.0085 | 0.2496 |
|  | White, Non-Hispanic | 0.0455 | 0.0085 | 0.0824 |
